# Acute kidney injury after treatment with arsenic trioxide 

**DOI:** 10.5414/CNCS111813

**Published:** 2025-12-11

**Authors:** Hatem Najar, Julia Evans, Sai Vedula, Gina Keiffer, Jingjing Zhang

**Affiliations:** 1 Division of Nephrology, Department of Medicine, Sidney Kimmel Medical College,; 2 Sidney Kimmel Medical College, and; 3 Department of Medical Oncology, Sidney Kimmel Comprehensive Cancer Center, Thomas Jefferson University, Philadelphia, PA, USA

**Keywords:** acute interstitial nephritis, acute kidney injury, acute promyelocytic leukemia, arsenic trioxide

## Abstract

All-trans retinoic acid (ATRA) and arsenic trioxide (ATO)-based therapy has become the standard of care in the treatment of acute promyelocytic leukemia (APL). While ATO nephrotoxicity has been rarely reported, data on the specific mechanisms and types of renal injury remain scarce. We present a case of acute kidney injury (AKI) superimposed on chronic kidney disease (CKD) induced by ATO therapy in a patient diagnosed with APL. Biopsy findings revealed acute interstitial nephritis on a background of IgA nephropathy. The AKI resolved after the discontinuation of ATO therapy and initiating steroid treatment. The kidney function remained stable at 6-month follow-up. This case highlights the potential renal complications associated with ATO therapy and highlights the importance of monitoring kidney function in patients undergoing this treatment.

## Introduction 

Acute promyelocytic leukemia (APL) is a rare subtype of acute myeloid leukemia characterized by the accumulation of abnormal promyelocytes in the bone marrow. This condition is marked by a specific chromosomal translocation involving the retinoic acid receptor alpha (RARα) gene, which leads to the formation of the PML-RARA fusion gene [1]. The combination of all-trans retinoic acid (ATRA) and arsenic trioxide (ATO) therapy has significantly transformed the treatment of APL, achieving remarkable long-term survival rates exceeding 90% without the need for cytotoxic chemotherapy [2]. 

Reports of ATO-induced nephrotoxicity are rare. In landmark randomized trials – the German Austrian and the Southwest Oncology studies – there were no reports of acute kidney injury (AKI) [2, 3]. There was one case of AKI in the ATRA-ATO arm in the AML17 study, however it is unclear whether this is associated with ATRA or ATO therapy [4]. Rare reports of arsenic-induced rhabdomyolysis have also been described [5]. In this case, we document a case of AKI attributed to ATO, characterized by biopsy-confirmed acute interstitial nephritis and tubular damage in a patient with underlying IgA nephropathy. 

## Case report 

**Figure 1.**
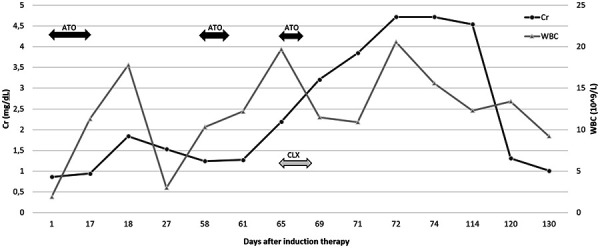
Timeline of arsenic use and corresponding serum creatinine and leukocyte count. ATO = arsenic trioxide; CLX = cephalexin; Cr = serum creatinine; WBC = white blood cells.

A 64-year-old male patient with a past medical history of essential hypertension, type 2 diabetes mellitus, chronic kidney disease stage 3a (baseline creatinine 1.2 – 1.3 mg/dL), cerebrovascular accident (CVA), seizure disorder and recently diagnosed APL, presented to our hospital with neutropenic fever. Two months prior to his admission, he was initiated on ATRA-ATO induction therapy and achieved morphologic remission on bone marrow biopsy after 24 days of daily therapy. His induction course was complicated by an acute CVA with resultant left-sided weakness and an AKI of unclear etiology that resolved after stopping ATO (Figure 1). 

On the day of admission, day 64 after induction, the patient was cycle 1, day 20 of consolidation treatment (day 20 of ATRA, day 5 of ATO). His list of medications included amlodipine, aspirin, gabapentin, levetiracetam, metformin, and sertraline. The patient was not on any herbal supplements. Blood tests revealed: serum creatinine (Cr) of 2.19 mg/dL; blood urea nitrogen (BUN) 29 mg/dL; potassium of 4.4 mEq/L; chloride of 104 mEq/L; albumin 3.1 g/dL; uric acid of 6.1 mg/dL; lactate dehydrogenase 197 U/L; aspartate aminotransferase 138 U/L, alanine aminotransferase 118 U/L; hemoglobin 8.0 g/dL; white blood cells 19,500 cells/mcL; platelets 239,000 cells/dL. Urinalysis revealed 2 + protein, 3+ blood, positive nitrites, 3+ leukocyte esterase, 182 red blood cells, > 182 white blood cells. 

The patient was diagnosed with an upper urinary tract infection (UTI), and the urine culture grew *klebsiella oxytoca*. He was treated with a 7-day course of cephalexin. Urine nitrites became negative after antibiotic therapy, but the patient had persistent pyuria, hematuria, and low-grade proteinuria (1+ on urinalysis). The patient’s kidney function continued to deteriorate over the course of hospitalization, despite fluid resuscitation and improved hemodynamic status. Renal ultrasound did not show evidence of obstruction. 

**Figure 2.**
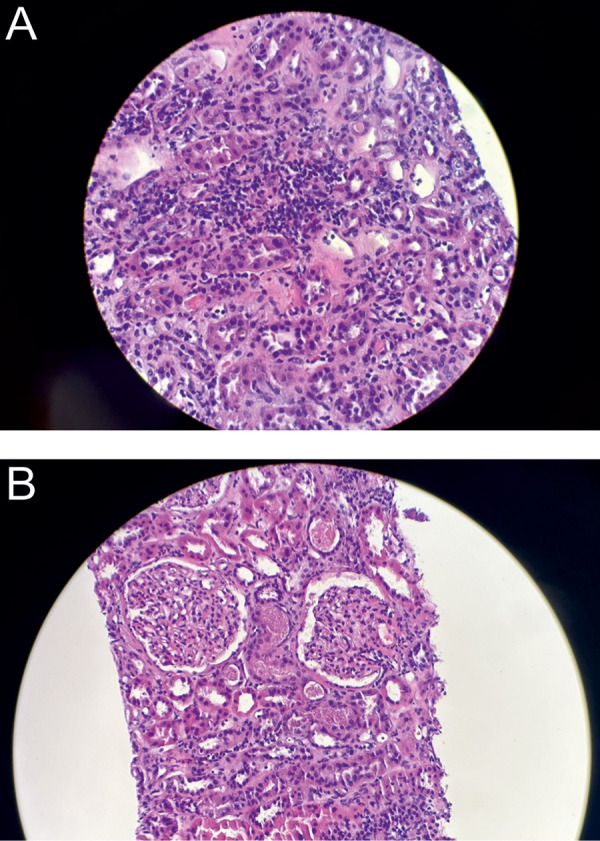
A: Light microscopy: H & E stain shows foci of inflammatory cell infiltration in the interstitial space with predominantly lymphocytes and scattered eosinophils. Tubules show mild epithelial swelling. B: Light microscopy: H & E stain shows mild increase of mesangial cells and matrix. There is no segmental sclerosis, endocapillary hypercellularity, and crescentic lesions.

Due to concerns for arsenic toxicity, the patient’s ATO was ultimately held while he continued ATRA monotherapy since admission. Serum creatinine peaked at 4.72 mg/dL (Figure 1). Considering the first episode of AKI during induction treatment that was resolved after stopping the ATO, the decision was made to perform a kidney biopsy, which revealed acute interstitial nephritis, moderate acute tubular injury, and IgA nephropathy (Figure 2). Light microscopy revealed > 50% mesangial hypercellularity with IF showing IgA 3+, C3 3+. EM showed minimal foot process effacement (10 – 15% of surface area). The GBM was intact throughout with thickening of the lamina densa. The electron-dense deposits were located in the mesangium. MEST-C score was M1 E0 S1 T0 C0. 

Based on the clinical picture and the pathological evidence, the patient was diagnosed with acute interstitial nephritis most probably secondary to ATO therapy, on a background of IgA nephropathy. The patient was subsequently initiated on prednisone taper therapy from admission day 14. Therapy was terminated earlier than the planned 3-month course after 5 weeks due to gastrointestinal bleeding. Cr returned to baseline at that time. At 6-month follow-up, Cr remained at baseline, Urinalysis revealed 1 + blood with < 1 RBC, with a UPCR of 0.34 mg/mg. 

## Discussion 

### Arsenic toxicity 

Our case illustrates acute interstitial nephritis most likely secondary to ATO, with moderate acute tubular damage on a background of IgA nephropathy; alternative contributors – sepsis, hypotension, or brief cephalexin exposure – were considered but did not align with the timing or clinical course of the AKI. The patient developed AKI during ATO therapy, evidenced by a rise in serum Cr from a baseline of 1.2 – 1.3 mg/dL to 4.72 mg/dL. The diagnosis of arsenic toxicity was supported by the temporal relationship between ATO therapy and the onset of renal dysfunction, improvement in renal function when ATO was held, and rapid recurrence of AKI upon ATO re-introduction. The patient’s kidney function improved significantly after discontinuation of ATO and initiation of prednisone 5 weeks after completion of steroids, the patient’s renal function ultimately returned to his pre-treatment baseline. We also believe that the impact of the UTI on the AKI episode is minimal, considering the absence of severe hemodynamic compromise. 

Data that specially evaluates renal complications of ATO therapy remains scarce. ATO, often combined with ATRA, has greatly improved treatment outcomes for APL, achieving over 90% long-term survival rates [2]. It is generally well-tolerated, but side effects may include QT prolongation, liver enzyme elevation, skin reactions, and gastrointestinal issues. The usual dose is 0.15 mg/kg/day until remission, with no set maximum dose. 

Studies done in both the Southwest Oncology Group and the German Austrian study groups showed no cases of renal failure [2, 3]. Moreover, in a phase 3 randomized controlled trial evaluating ATO and ATRA treatment for APL, no difference of raised Cr was observed between the ATO and control groups [4]. More recently, in a retrospective study of 108 APL patients treated with ATO-based therapy, 6 cases of clinically significant AKI were identified without alternative cause, no kidney pathology was reported [6]. Multivariate analysis found that only ATO dose was a significant predictor of AKI of unknown etiology [6]. 

Historical studies have linked acute arsenic poisoning in dogs with acute tubular necrosis, cortical hemorrhages, and glomerular sclerosis [7]. In vitro studies suggest arsenic exacerbates inflammation, oxidative stress, and endothelial dysfunction. In an in-vitro study evaluating sub-chronic ATO exposure on mice, Li et al. [8] showed that ATO induces oxidative DNA damage in kidney tissues, primarily through the generation of reactive oxygen species (ROS). This oxidative stress leads to significant increases in 8-hydroxy-2-deoxyguanosine (8-OHdG) levels, a marker of oxidative DNA damage, particularly in the epithelial cells of the proximal convoluted tubules and the podocytes. The resulting cellular damage manifests as histopathological changes, including cellular swelling, tubular dilatation, and lymphocytic infiltration, which could be indicative of acute tubular damage and interstitial inflammation. These findings suggest that the nephrotoxic effects of arsenic are closely linked to its ability to induce oxidative stress and subsequent DNA damage, leading to structural and functional impairments in the kidney [8]. 

More recently, numerous epidemiologic studies have also demonstrated a positive association between chronic environmental arsenic exposure and the prevalence of chronic kidney disease (CKD) [9, 10]. In a systematic review on the effects of arsenic exposure on CKD, Zheng et al. [10] highlighted a generally positive association between arsenic exposure and CKD. High arsenic levels, particularly in drinking water, have been linked to increased risks of albuminuria and proteinuria, both markers of kidney damage [11, 12]. 

The severity of our patient’s kidney dysfunction can also be attributed to a synergistic effect of arsenic toxicity and IgA nephropathy. As explained earlier, the toxic effect of arsenic induces tubular damage and interstitial inflammation [8]. Concurrently, IgA nephropathy, characterized by deposition of IgA immune complexes in the mesangium of glomeruli, induces local inflammation and mesangial hypercellularity, hardly affecting the kidney function in the absence of endothelial proliferation and crescent formation [13]. It is possible that the inflammatory response from IgA nephropathy, coupled with the nephrotoxic effects of ATO, created a compounded effect, resulting in a more severe renal dysfunction that neither condition alone would have caused [14]. 

### IgA nephropathy 

Our patient was also diagnosed with IgA nephropathy with the pathology revealing mesangial expansion by an increase in the matrix cells, and the presence of mesangial electron-dense deposits and 3+ IgA on immunofluorescence staining. The development of microscopic hematuria and proteinuria is consistent with the diagnosis of IgA nephropathy. 

Infections such as UTIs present in our patient could lead to a temporary exacerbation of underlying primary IgA nephropathy. 

However, the coexistence of acute APL raises the possibility of secondary IgA nephropathy as well. A systematic review of secondary IgA nephropathy case reports by Tota et al. [15] showed liver cirrhosis as the most common trigger. Moreover, secondary IgA nephropathy has been associated with various oncological conditions such as IgA myeloma, renal cell carcinoma, Hodgkin’s and non-Hodgkin’s lymphoma, and lung cancers [16]. A literature review revealed no known case reports of APL and secondary IgA nephropathy but case reports of non-Hodgkin’s lymphoma and secondary IgA nephropathy [17]. 

Secondary and primary IgA nephropathy share similar pathogenesis regarding the galactose-deficient O-glycans of the circulating IgA1 and glomerular deposition of IgA1 [18]. The exact pathophysiologic mechanism remains unclear. It also remains unclear if there is a direct mechanism or if the renal findings could be a paraneoplastic involvement as reported in some case reports of Hodgkin’s disease in children [19]. Further studies are needed to understand the mechanisms between lymphoma and secondary IgA nephropathy. 

At this point, we will not be able to know if the patient’s IgA nephropathy is primary or secondary. It would be interesting to see the resolution of IgA nephropathy after achieving remission of leukemia, but there is no indication to re-biopsy the patient up to this point. We believe the main culprits of AKI in our patient are acute interstitial nephritis and tubular damage. The quick recovery of kidney function is consistent with the above assumption. At 6-month follow-up, Cr remained at baseline, urinalysis revealed 1 + blood with < 1 RBC, with a UPCR of 0.34 mg/mg, further confirming our hypothesis. 

This case underscores the need for vigilance for rare renal complications during ATO therapy and highlights the value of timely biopsy and steroid treatment in preserving renal function. 

## Authors’ contributions 

Julia Evans, Gina Keiffer, Jingjing Zhang collected clinical data. 

Hatem Najar, Sai Vedula, Gina Keiffer, Jingjing Zhang wrote the discussion. 

Hatem Najar modified all the drafts of the text through out, supervised the project. 

All authors reviewed the manuscript. 

## Funding 

This work did not receive any specific grant or funding.

## Conflict of interest 

The authors have no conflict of interest to declare.
